# Protective measures for patients with advanced cancer during the Sars-CoV-2 pandemic: Quo vadis?

**DOI:** 10.1007/s10585-021-10083-1

**Published:** 2021-03-23

**Authors:** P. Ivanyi, T. Park-Simon, H. Christiansen, R. Gutzmer, A. Vogel, M. Heuser, H. Golpon, P. Hillemanns, J. Haier

**Affiliations:** 1 Department of Hematology, Hemostasis, Oncology and Stem Cell Transplantation, Hannover Medical School, 6860, Carl–Neuberg Strasse 1, 30625 Hannover, OE Germany; 2 Department of Gynecology and Obsterics, Hannover Medical School, Hannover, Germany; 3 Department of Radiotherapy and Special Oncology, Hannover Medical School, Hannover, Germany; 4 Department of Dermatology and Allergy, Skin Cancer Center Hannover, Hannover Medical School, Hannover, Germany; 5 Department of Gastroenterology, Hepatology and Endocrinology, Hannover Medical School, Hannover, Germany; 6 Department of Pulmonology, Medizinische Hochschule Hannover, Hannover Medical School, Hannover, Germany; 7 Comprehensive Cancer Center Niedersachsen (CCC-N), Hannover Medical School, Hannover, Germany; 8 Immuncooperative Oncology Group (ICOG) of CCC-N, Hannover Medical School, Hannover, Germany

**Keywords:** Sars-CoV-19, COVID-19, Metastasis, Treatment, Cancer

## Abstract

Cancer patients represent a vulnerable cohort during the Sars-CoV-2 pandemic. Oncological societies have generated a plethora of recommendations, but precise instructions about routine oncological procedures remain scarce. Here, we report on local COVID-19 protection measures established in an interdisciplinary approach at a tertiary care center during the first wave of the pandemia in Germany. Following these measures, no additional morbidity or mortality during oncological procedures was observed, and no nosocomial infections were registered. However, Validation of our measures is outstanding and regional SARS-CoV-2 prevalence was low. However, specific oncological measures might be important to ensure optimal oncological results, especially for advanced cancer stages during this and future pandemia. In the future, communication about these measures might be crucial to a cancer patient´s assigned network to reduce the danger of excess mortality within the second wave of the COVID-19 pandemic.

**Supplementary Information:**

The online version contains supplementary material available at 10.1007/s10585-021-10083-1.

## Commentary

SARS-CoV-2 spread from Wuhan all over the world, ultimately causing a global pandemia affecting several aspects of medicine, as well as oncology. Patients and physicians have had to face several new challenges [1]. Although the number of new infections in Germany was low within the first half of 2020, lack of broad immunity towards SARS-CoV-2 within society requires SARS-CoV-2 to be part of everyday clinical practice. Nonetheless, SARS-CoV-2 has again become particularly important during the ongoing second wave in Germany. Oncological diseases and therapies could cause additional morbidity and mortality in the context of the SARS-CoV-2 pandemic, which might selectively affect patients with advanced or metastatic diseases. Both pandemia-related logistical bottlenecks and SARS-CoV-2 need to be considered when counseling patients. Conversely, excessive protective measures, or fears among patients could lead to increased mortality in cancer care [2–4]. Finally, it is a matter of debate whether cancer associated immunological incompetence, or immunological effects of cancer therapies are protective or are accelerating factors towards SARS-CoV-2 associated lung failure, most likely based on a hyperinflammatory mechanism [5].

Preliminary data suggest that infection rates with SARS-CoV-2 are comparable between cancer and non-cancer patients [6–8]. At present, 20% of SARS-CoV-2 cases are assumed to be severe, and at least 4% demand intensive care support [9, 10]. Age, as well as concomintant cardiovascular diseases, are major risk factors for a serious course of this infection [11]. Experience from comparable constellations with community-acquired respiratory viruses suggests that cancer patients are at considerable risk of SARS-CoV-2 related mortality of up to 25% [12–14]. Preliminary data of SARS-CoV-2 are suggestive of an increased risk in cancer patients with high rates of severe courses (up to 39%) and mortality (up to 29%). Further on, oncological therapies might have an impact on both parameters [6, 8]. These concerns are supported by prospective data from the CCC-19-, Teravolt-, as well as U.K. registries, recently presented at ASCO and ESMO. However, the majority of current publications is limited in impact, due to methodological restrictions, special aspects of a pandemia, selection criteria, regional differences, or missing peer review as well as small numbers of cases [15–17]. It is currently elusive to what extent the symptoms of SARS-CoV-2 disease are similar among cancer and non-cancer patients. Also, an overlap of symptoms of SARS-CoV-2 and cancer treatment related adverse events can be assumed. Both considerations, rises concerns, that differential diagnosis might cause additional confusions during the SARS-CoV-2 pandemic [18]. Ultimately it needs to be clarified, that the oncological care intensity and measures need to be adapted to the extent of regional outbreak. on the pandemic regional level; considerations in regions with a high SARS-CoV-2 prevalence, possibly accompanied by medical and personal resource bottlenecks, seems to differ from regions with a low prevalence The needs and requirements obviously differ from regions with low SARS-CoV-2 prevalence to regions with a high prevalence that possibly face medical and personal resource bottlenecks [19].

Oncologic practitioners are challenged to manage under- and over-treatment in the everyday world, and even more so within a pandemic Further on, cancer patients are at particular risk of excess mortality, as well as increased SARS-CoV2-related mortality [2]. The likelihood of SARS-CoV-2-related death is usually lower than the risk arising from metastatic disease; however, a severe SARS-CoV-2 course can lead to the premature death of these patients [5]. Several recommendations exist about treatment during the current pandemic [20–22]. However, from the view of evidence-based medicine, these guidelines present rather pragmatic perspectives of risk/benefit evaluation. So far, there are hardly any procedural suggestions available. Here, we present an interdisciplinary consensus as a result of communication with practitioners at the Comprehensive Cancer Center Lower Saxony (CCC-N) and the local university hospital SARS-CoV-2 task-force. Therein, based on weekly phone conferences, local pandemic assumptions, local experience and published data, a plethora of measures were discussed (structured panel discussion) and considered to be integrated into daily clinical routine. Discussed measures considered several aspects: administration, cancer care plans, patient-directed, hospital preparedness, employee directed, as listed in Table 1. Although the majority of measures addressed risk reduction for SARS-CoV-2 exposure, either to patients or to staff, adjusting cancer care plans is challenging and needs to be individualized. In our experience, reflecting routine oncological decision steps in an interdisciplinary manner is key to achieving optimized pandemic adapted oncological treatment pathways. (Supp. Fig. S1, Fig. S2, Supplemental Fig. S3, S4).

**Table 1:** Summary of SARS-Co-V2 cancer measures at the comprehensive cancer center lower saxony in hannover (CCC-N)

Administration	Inauguration of SARS-CoV-2-Task-Force (whole hospital, all disciplines) Inauguration of Cancer-Task-Force (weekly phone calls)
Logistics	Securing of drug supply chains (Pharmacy) Defining access pathways for hospitalized patients and outpatients, preventing contact of SARS-CoV-19 and cancer patients IT-based interdisciplinary phone conferences Reducing tumor board participants to a minimum IT-based teaching for medical students Measures for ensuring distances in waiting and infusion areas, Personal protection equipment (PPE) instruction for all visitors accessing outpatient area Stringent restriction of entry for all non-patients or employees
Cancer care	Interdisciplinary definition of critical steps in oncological treatment algorithms (Fig. 1, Supp. Figs. S1-3) Postponing highdose-chemotherapy and allogeneic PBSCT in case of deep remission
Patient-directed	Evaluation of SARS-CoV-2 symptoms prior to contact to inpatient or outpatient clinics (phone based, 1 day prior to hospital visit for all patients) Clinical and temperature evaluation at admission to hospital or entry of infusion area, SARS-Co-V-2 screening in suspicious cases
Hospital-preparedness	Establishment of standard operating procedures for cases of suspected or confirmed SARS-CoV-2 infection Establishment of isolation wards Expanding palliative care capacities
Employees-directed	Provision of risk adapted PPE for high-risk and low risk areas areas Ongoing education in hygiene by department of hygiene/infectious diseases Self-screening for infectious complications

**Fig. 1:**
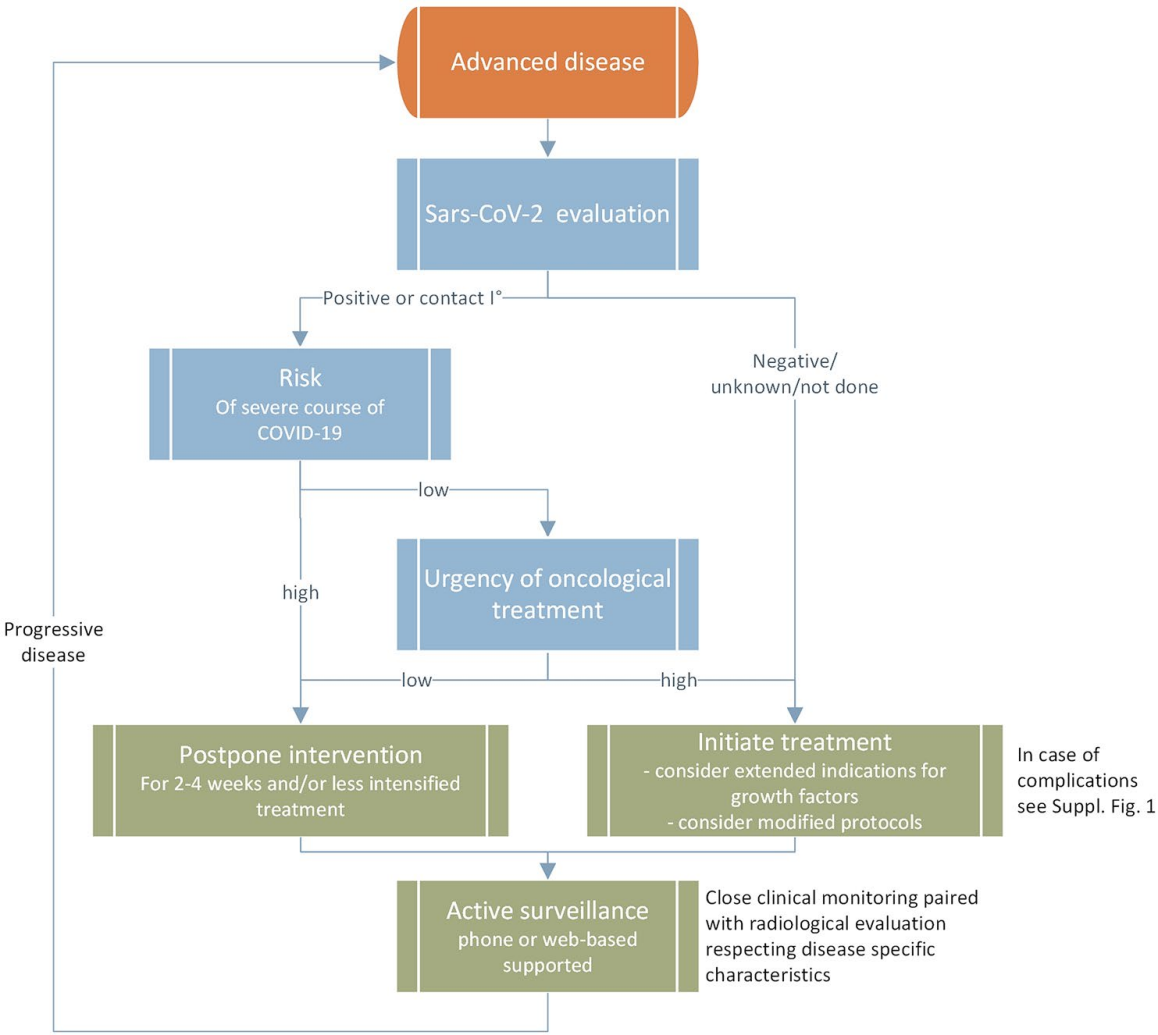
Pandemic-adapted oncological consideration algorithm for advanced disease status

In general, procedural questions arise at several oncological decision steps, requiring interdisciplinary exchange (Fig. 1, Supp. Fig. S1-3). With regard to oncological therapy in the metastatic setting, neoadjuvant, adjuvant, or palliative, primarily the patient´s risk of acquiring or becoming positive for SARS-CoV-2 needs to be assessed (Fig. 1). In addition to a sufficient medical history and examination with regard to SARS-CoV-2 exposure and symptoms, potentially a methodology-adequate a SARS-CoV-2 test must be considered in accordance with local regulations. SARS-CoV-2 testing is of particular importance when considering in hospital-procedures, rather than out-patient procedures, since nosocomial SARS-CoV-2-infections were described in China’s cancer cohorts [6, 20]. A positive SARS-CoV-2 test result justifies in most cases a postponement of treatment initiation for up to four weeks, although oncological remission pressure, critical anatomical aspects of metastasis and underlying tumor biology need to be considered (Fig. 1). In case of a negative SARS-CoV-2 test, and prior to therapy, risk factors for a severe course of a potential SARS-CoV-2 infection (e.g. age, chronic heart diseases, lymphopenia, D-dimer elevation, immunosuppression) should be evaluated and balanced with the planned anti-cancer treatment (Fig. 1) (23). operability, local control rate, the individualization of post-neoadjuvant therapy and overall survival. (23); in individual cases, neoadjuvant or adjuvant therapy might be omitted (see Supplement Figs. S1, S2, and S3).

Beyond SARS-CoV-2 evaluation, once the decision is made in favor of anti-cancer therapy, the question of treatment urgency needs to be addressed. Apart from objective remission pressure, there are little data on the benefit of immediate versus delayed treatment initiation, in particular in the metastatic setting. Therefore, depending on local infection rates, availability of resources and an individual patient´s risk for a severe course of a potential SARS-CoV-2 infection must be weighed against the benefits of immediate treatment initiation. Drug holidays or watchful waiting might be considered as alternatives to active treatment, especially in the advanced disease cases, during a high SARS-CoV-2 prevalence, and in high risk patients (Fig. 1) [23]. At the start of palliative therapy due to advanced or metastatic disease status, the possibility of a less intensive therapy should be discussed in the first-line setting, as well as throughout sequencial palliative therapy, in particular once remission pressure is considered to be low. Here, the ESMO prioritization between high, medium and low priority therapies appears adequate for everyday use, when addressing the question of which regimen to select [20]. For example, gemcitabine/abraxane instead of FOLFIRINOX in advanced pancreatic cancer, or de-escalation of doxorubicin/ifosfamide to doxorubicin in soft-tissue sarcoma, or early de-escalation of inductive immuno-oncological combination (CTLA-4/PD-1) to PD-1 monotherapy could be considered. Response rates, as well as overall survival, main drivers of standard of care definitions, should be critically weighed against rates of adverse events and hospitalizations during a pandemic.

SARS-CoV-2 represents a new relevant differential diagnosis for infectious complications during therapy. The management of febrile neutropenia according to the S3 guideline and the local Sars-CoV-2 standard appears appropriate (Supp. Fig. S1). A shift in chemotherapy e.g. to four weeks and continuation of systemic therapy after recovery of lymphopenia or granulopenia seem justifiable (see Supp. Fig. S4). In principle, an earlier preventive use of hematolymphopoietic growth factors is another possibly useful supportive measure, considering potential bottlenecks in blood supply [23]. In the case of follow up, IT-based applications may help to reduce the possibility of SARS-CoV-2 exposure and should be considered [20, 24].

Radiotherapy usually consists of a longer serial therapy period without interruption (Supp. Fig. S2). This type of therapy needs to remain feasible during a pandemic, in particular in the curative setting, or once tumor burden qualifies a patient for radiotherapy. Nevertheless, depending on the risk posed by the pandemic situation, the need for therapy must be checked critically in an interdisciplinary manner. Also dosing has to be critically evaluated. In order to keep the total treatment time as short as possible, hypofractionated radiation could be used preferentially during a pandemic, in accordance with the current recommendations and level of evidence (see Supp. Fig. S3) [25].

Although considerations were made mainly for the advanced or metastatic setting, application to the limited diesease setting is also feasible, as illustrated in Supplemental Figs. S3 and S4.

In summary, the Sars-CoV-2 pandemia poses a major challenge to interdisciplinary oncology and a strong interdisciplinary discussion – always essential in oncology—is necessary for now and in the near future to ensure a more individualized approach to treatment decisions. Due to our experience, oncology in its best form according to interdisciplinary consensus is essential more than ever. due to our experience. The procedural considerations presented here, resulting from an ongoing interdisciplinary structured discussion, enabled our center to continue oncological therapies in the CCC-N without relevant additional Sars-CoV-2-related morbidity or mortality. No nosocomial infections were so far observed. Doubtless, this has to be critically reflected by the fact that local SARS-CoV-2 infections rates were low during the first wave of the pandemic and resource bottlenecks did not define our daily work. However, our measures have not yet been stress tested.

Under or over-treatment, as well as increased excess mortality, must also be avoided in future pandemic phases. The cumulative oncological network knowledge and data expertise, both in the context of interdisciplinary tumor conferences and in national network structures such as CCCs and NCTs, appear to be required and useful to optimally adjust and communicate the individual therapy algorithm during the pandemic. This is essential for the foreseeable future. Ultimately, the current SARS-CoV-2 pandemc is an opportunity to generate evidence to better define needs of oncological patients for future pandemia, which hopefully won’t appear to soon.

## Supplementary Information

Below is the link to the electronic supplementary material.

Supplementary file1 (JPG 575 KB)

Supplementary file2 (JPG 590 KB)

Supplementary file3 (JPG 286 KB)
