# Supplementary figures and images for: Protective measures for patients with advanced cancer during the Sars-CoV-2 pandemic: Quo vadis?
Source: Clin Exp Metastasis. 2021 Mar 23;38(3):257–61. doi: 10.1007/s10585-021-10083-1 (PMC7987238; doi:10.1007/s10585-021-10083-1)

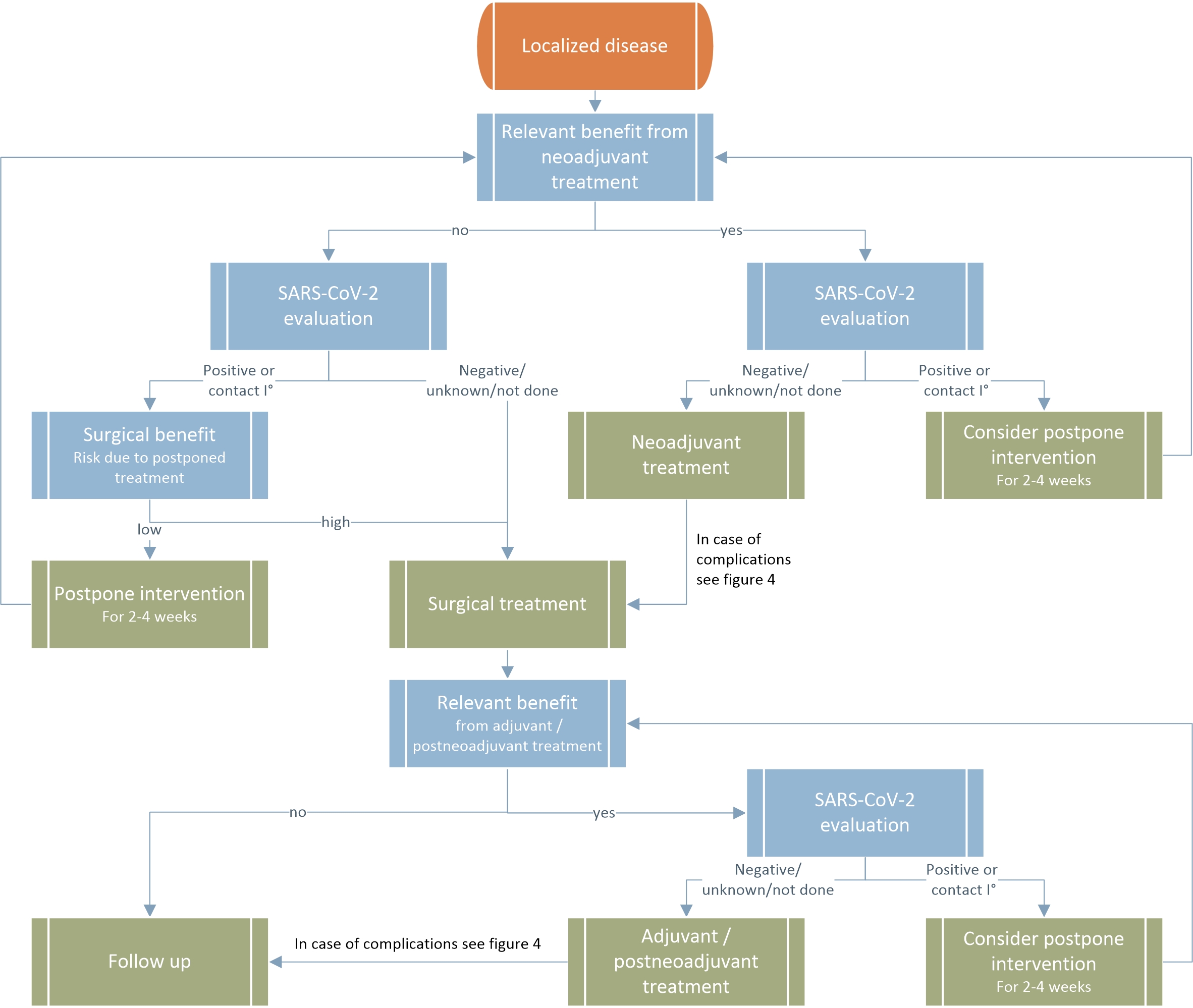

Supplement: Supplementary file 1 — Supplementary file1 (JPG 575 KB) [file 10585_2021_10083_MOESM1_ESM.jpg]

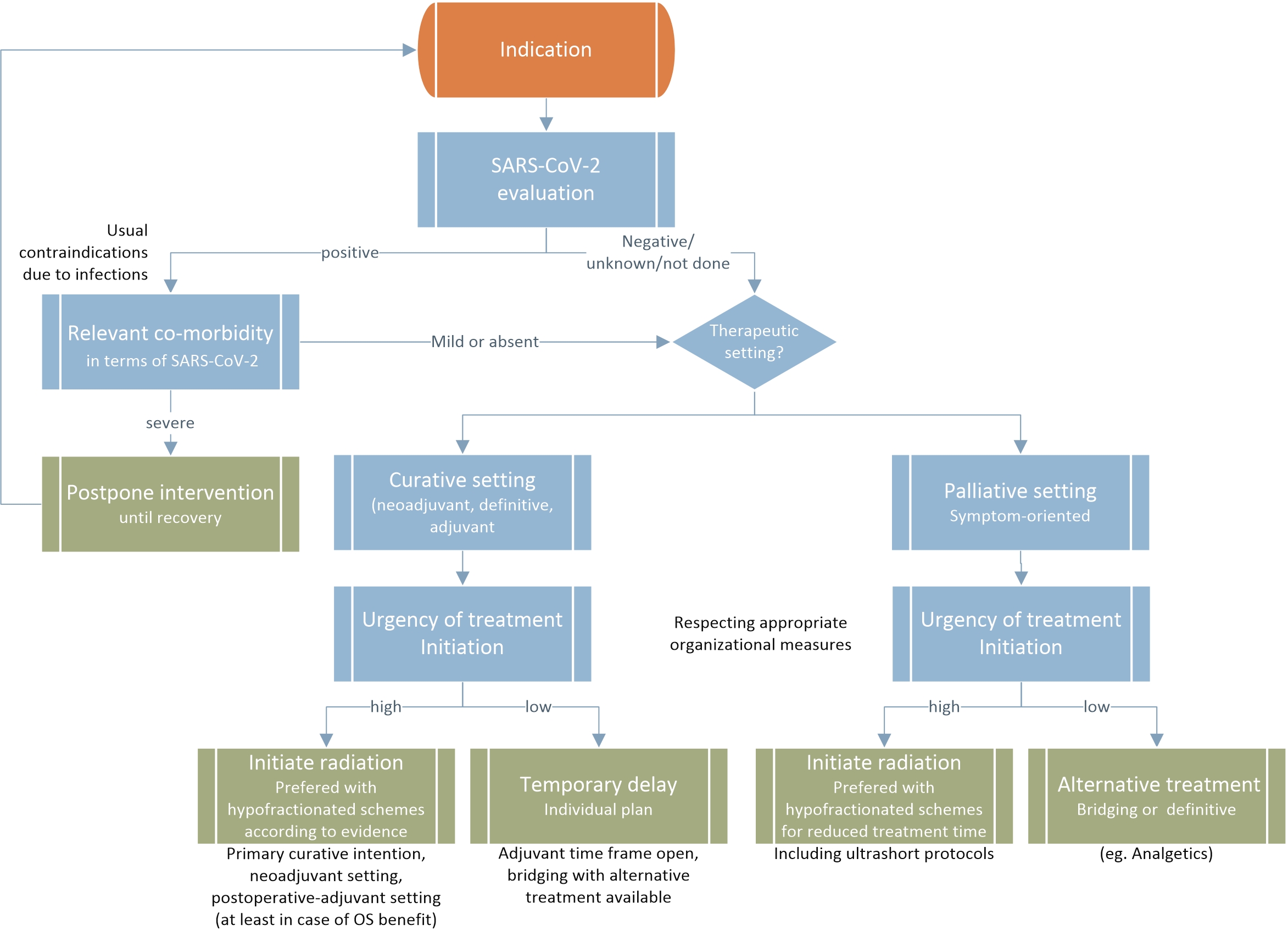

Supplement: Supplementary file 2 — Supplementary file2 (JPG 590 KB) [file 10585_2021_10083_MOESM2_ESM.jpg]

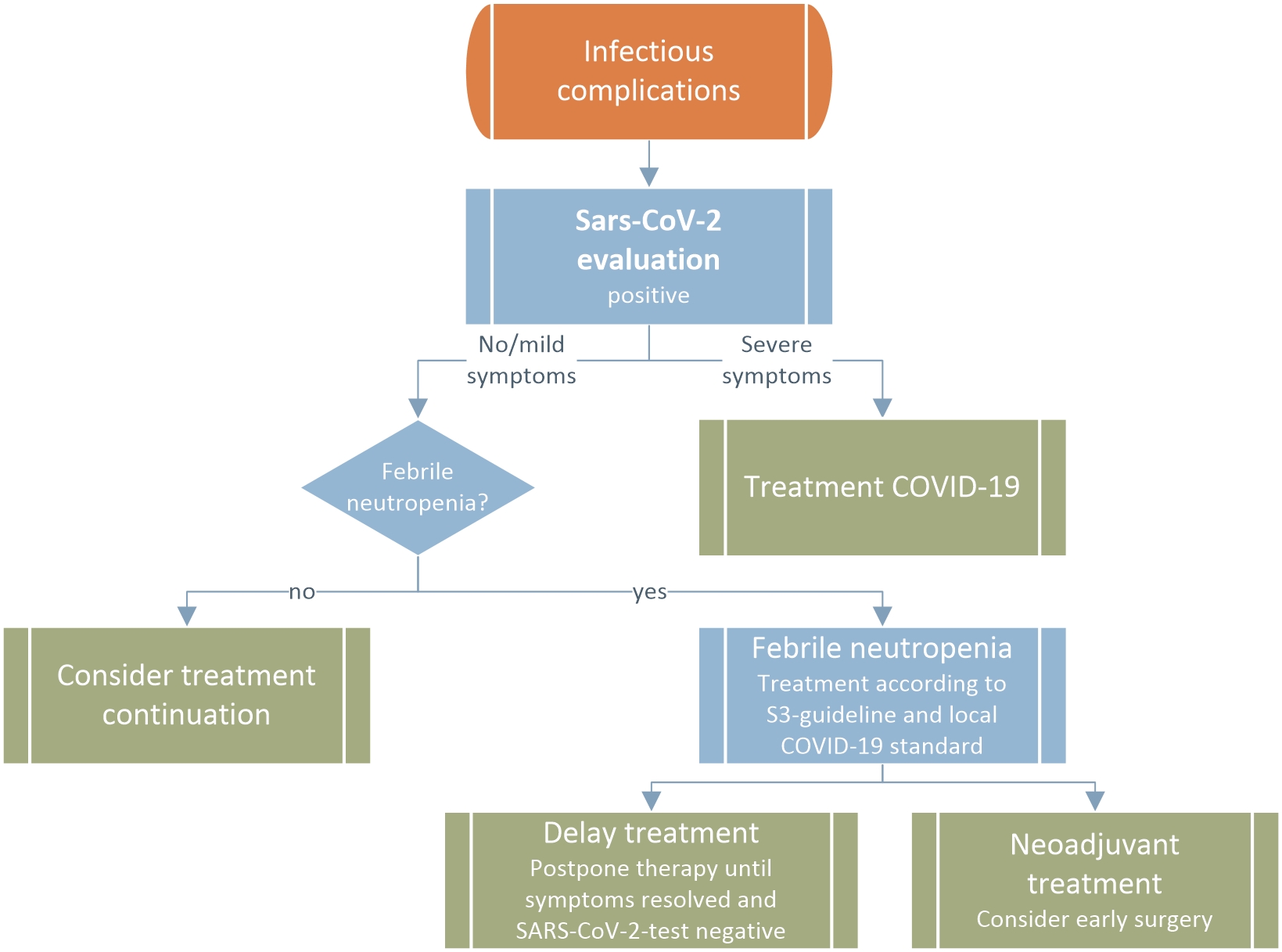

Supplement: Supplementary file 3 — Supplementary file3 (JPG 286 KB) [file 10585_2021_10083_MOESM3_ESM.jpg]
